# Supplementary material for: A novel cloud removal method by fusing features from SAR and neighboring optical remote sensing images
Source: PLoS One. 2025 Sep 23;20(9):e0332898. doi: 10.1371/journal.pone.0332898 (PMC12456809; doi:10.1371/journal.pone.0332898)
Supplement: Attachment — Submitted filename: Response to Reviewers.docx [file pone.0332898.s002.docx]

Dear Editor and Reviewers,

We sincerely appreciate your positive and constructive comments on our paper. Based on the suggestions from the editor and reviewers, we have carefully revised the manuscript and marked the changes using the track changes mode.

Below, we provide a detailed list of the revisions made compared to the original submission. We are truly grateful for all the valuable feedback from the reviewers and the editor.

**Paper:** A novel cloud removal method by fusing features from SAR and neighboring optical remote sensing images

**Response to the Editor:**

Response to the Editor's Comments

1. When submitting your revision, we need you to address these additional requirements. Please ensure that your manuscript meets PLOS ONE's style requirements, including those for file naming. 
**Response:** We adjusted the manuscript to comply with PLOS ONE's formatting requirements, including file naming conventions.

2. Please note that PLOS ONE has specific guidelines on code sharing for submissions in which author-generated code underpins the findings in the manuscript. In these cases, we expect all author-generated code to be made available without restrictions upon publication of the work.
**Response:** In accordance with the guidelines of PLOS ONE, we uploaded all author-generated code to the figshare database and confirmed that our code adhered to best practices. The DOI for the code is: 10.6084/m9.figshare.28623698. We ensured that the code was publicly accessible without any restrictions for use and validation by other researchers.

3. We note that the grant information you provided in the ‘Funding Information’ and ‘Financial Disclosure’ sections do not match. 
**Response:** Thank you for pointing this out. There are three grants supporting this study. Since the third grant name could not be found in the submission system, only the first two grants were entered. Please refer to the following grant information as the correct one. We provided the correct funding information in the "Funding" section at the end of the Revised Manuscript with Track Changes file: This research was funded by the National Natural Science Foundation of China (No. 41671514), the Natural Science Foundation of Henan Province (No. 162300410122), and the Surveying and Mapping Science and Technology "Double First-Class" Discipline Creation Project (No. GCCYJ202409, No. BZXCG202403).

4. We note that your Data Availability Statement is currently as follows: All relevant data are within the manuscript and its Supporting Information files. Please confirm at this time whether or not your submission contains all raw data required to replicate the results of your study. Authors must share the “minimal data set” for their submission.
**Response:** Thank you for pointing this out. We uploaded the minimal data set used in our experiments to the figshare database to support the replication of our study's results. The DOI for the data set was: 10.6084/m9.figshare.28623698.

5- We require you to either present written permission from the copyright holder to publish these figures specifically under the CC BY 4.0 license, or remove the figures from your submission: You may seek permission from the original copyright holder of Figure 6 to publish the content specifically under the CC BY 4.0 license.

**Response:** Thanks. The map images in Figure 6 were based on high-resolution imagery from China’s Gaofen satellites, which had been purchased by our research team. We confirmed that the copyright for these images allowed for their public use and dissemination, including publication under the CC BY 4.0 license for your journal. We had also signed the content-permission form and uploaded it as an attachment to ensure compliance with the CC BY 4.0 license.

6- We note that Figures 7, 8, 9, 10, 11, 12, 13 and 16 in your submission contain copyrighted images. All PLOS content is published under the Creative Commons Attribution License (CC BY 4.0), which means that the manuscript, images, and Supporting Information files will be freely available online, and any third party is permitted to access, download, copy, distribute, and use these materials in any way, even commercially, with proper attribution. We require you to either present written permission from the copyright holder to publish these figures specifically under the CC BY 4.0 license, or remove the figures from your submission:

**Response:** The copyright status of the map images in Figures 7–13 and 16 was the same as Figure 6, and we followed the same approach.

7- Please review your reference list to ensure that it is complete and correct. If you have cited papers that have been retracted, please include the rationale for doing so in the manuscript text, or remove these references and replace them with relevant current references. Any changes to the reference list should be mentioned in the rebuttal letter that accompanies your revised manuscript. If you need to cite a retracted article, indicate the article’s retracted status in the References list and also include a citation and full reference for the retraction notice.

**Response:** We carefully checked the reference list and confirmed that no retracted papers were cited in our manuscript. In addition, we revised the reference format to comply with PLOS ONE's journal standards and ensured that the reference list was complete and accurate. In response to Reviewer 2's third comment, we added reference [19] in line 97 of the introduction. Additionally, we removed the original references [26], [27], [28], and [29], and added new references [26] and [27] specifically for the SAM index and mSSIM index.

**Responses to the reviewers:**

Responses to the Reviewer’s Comments

**Reviewer 1**

1. Page 22, Datasets. It would be very useful if this algorithm is applied to a large area, for example an area of 30 km by 30 km. Can you do this experiment?

**Response:** Thank you for your suggestion. In our original approach, we divided a 7.3 km × 7.3 km remote sensing image into 128 × 128 pixel (meter) patches and trained the CGAN model on a laptop with an 8GB GPU, where the maximum memory usage reached 5.9GB. Training for 50 epochs and making predictions took a total of 24 minutes. We then conducted experiments on a high-performance computer with a 16GB GPU, dividing a 30 km × 30 km remote sensing image into 128 × 128 pixel (meter) patches. The maximum memory usage reached 14.9GB, and the experiment was successfully executed, yielding promising results. The total time for training 50 epochs and prediction on this large-area image was 19 minutes. These findings demonstrate that our method can effectively process large-area remote sensing images.

2. Page 32, Discussion. Discuss the limitations of your method and draw some future directions.

**Response:** We moved the discussion of the limitations and future directions from the end of the Conclusion section to Section 4.4 of the Discussion and expanded this section accordingly. The revised content can be found in Section 4.4.

=============================================================

**Reviewer2**

1. The abstract should include a reflection of the specific indicators and numerical values of the experimental results.

**Response:** Thanks. We revised the abstract to include specific experimental results as suggested. The updated version now presents quantitative indicators such as RMSE, SAM, mSSIM, and CC. Please refer to the abstract for details.

2. At the end of the abstract, a sentence should be provided stating the significance of this study for the field.

**Response:** Thank you for your suggestion. We revised the final part of the abstract accordingly. The updated version now concludes with the following statement: " The effectiveness of the proposed method will improve the quality of cloud removal in optical remote sensing images."

3. The sentence 'It can be confidently stated that' in line 97 of the introduction is not suitable for use in the paper. Please cite appropriate references as support.

**Response:** We revised the original sentence to "Regardless of how complex the terrain conditions of a region were, or how high the temporal resolution requirements for the studied objects in optical remote sensing images might have been, it was always possible to find cloud-free optical remote sensing images for areas that were cloud-covered." Additionally, we supplemented reference [19] to support the statement.

4. Before introducing the contribution of this article, provide a more clear transition.

**Response:** Thank you for your suggestion. We revised the content as follows: " However, existing methods have yet to effectively integrate SAR image features with neighboring optical image features for cloud removal in optical remote sensing images, thus limiting the enhancement of cloud removal performance. To tackle this issue, this study proposes a novel cloud removal method that integrates SAR and neighboring optical remote sensing image features."

5. Restructure Key Contributions: It may be worth condensing these points into clear contributions and then elaborate on them further in the document.

**Response:** Thanks. We made the corresponding revisions as suggested, and the updated key contributions can be found in the last section of the Introduction.

6. It is necessary to improve the resolution of Figure 6. In addition, the clarity of the experimental results figures in the article is poor. Please improve the figures quality appropriately，you can convert the image into a vector image in SVG format.

**Response:** Thanks. We revised all the images by converting them to TIF format with an improved resolution of 330 dpi and uploaded them as attachments.

7. It is necessary to discuss the unpredictable results or patterns encountered in this study, as well as the limitations of this research.

**Response:** Thanks. We moved the discussion of the limitations and future directions from the end of the Conclusion section to Section 4.4 of the Discussion and expanded this section accordingly. The revised content can be found in Section 4.4.
