# Supplementary material for: A novel cloud removal method by fusing features from SAR and neighboring optical remote sensing images
Source: PLoS One. 2025 Sep 23;20(9):e0332898. doi: 10.1371/journal.pone.0332898 (PMC12456809; doi:10.1371/journal.pone.0332898)
Supplement: Attachment — Submitted filename: Response_to_Reviewers_auresp_2.docx [file pone.0332898.s003.docx]

Dear Editor and Reviewers,

We sincerely appreciate your positive and constructive comments on our paper. Based on the suggestions from the editor and reviewers, we have carefully revised the manuscript and marked the changes using the track changes mode.

Below, we provide a detailed list of the revisions made compared to the original submission. We are truly grateful for all the valuable feedback from the reviewers and the editor.

**Paper:** A novel cloud removal method by fusing features from SAR and neighboring optical remote sensing images

**Response to the Editor:**

Response to the Editor's Comments

1. If you would like to make changes to your financial disclosure, please include your updated statement in your cover letter. 
**Response:** Thank you for the reminder. Our financial disclosure remains unchanged. The study was supported by the National Natural Science Foundation of China (No. 41671514), the Natural Science Foundation of Henan Province (No. 162300410122), and the Surveying and Mapping Science and Technology "Double First-Class" Discipline Creation Project (No. GCCYJ202409, No. BZXCG202403).

2. If applicable, we recommend that you deposit your laboratory protocols in protocols.io to enhance the reproducibility of your results. Protocols.io assigns your protocol its own identifier (DOI) so that it can be cited independently in the future.
**Response:** Thank you for the suggestion. Our data, code, and experimental procedures have already been deposited on the Figshare platform to ensure transparency and reproducibility. The DOI is: https://doi.org/10.6084/m9.figshare.28623698.v1.

3. Please review your reference list to ensure that it is complete and correct. If you have cited papers that have been retracted, please include the rationale for doing so in the manuscript text, or remove these references and replace them with relevant current references. Any changes to the reference list should be mentioned in the rebuttal letter that accompanies your revised manuscript. If you need to cite a retracted article, indicate the article’s retracted status in the References list and also include a citation and full reference for the retraction notice.
**Response:** TThank you for the suggestion. In response to the reviewers’ comments, we have added the following references to the revised manuscript: references [6], [7], [8], [13], [14], [22], and [30]. We have carefully reviewed the entire reference list to ensure that it is complete and accurate. No retracted articles have been cited in our manuscript.

**Responses to the reviewers:**

Responses to the Reviewer’s Comments

**Reviewer3**

We sincerely apologize for the discrepancy between the line numbers mentioned in the reviewers’ comments and those in our manuscript. To help accurately locate the corresponding revisions, we have added the updated line numbers from the resubmitted manuscript in each response.

1. L53 to L55: It is proven that… here you need a reference containing this proof.

**Response:** Thank you for your comment. We have revised the sentence in Lines 62–63 and added appropriate supporting references [6], [7], and [8] to provide evidence for the statement.

2. L54: Can you elaborate a bit (here or in any suitable part) about the types of cloud the proposed methodology.

**Response:** Thank you for your suggestion. We have elaborated on the types of clouds that the proposed methodology can handle in Lines 106–111 of the revised manuscript, specifically within the sentence beginning with “Compared with traditional methods that...”. We clarified that the method is applicable to both thin clouds and thick clouds.

3. L56: Give an example of what kind of auxiliary data are needed.

**Response:** Thank you for your comment. The types of auxiliary data required—mainly cloud-free optical images from other time phases or data from other sensor types—have already been clarified in the later sections of the manuscript where the introduction and methods are described.

4. L64: One general comment, remote sensing appears many times. Use abbreviation RS instead.

**Response:** Thank you for the helpful suggestion. Except for the abstract, we have replaced all occurrences of “remote sensing” with the abbreviation “RS” throughout the manuscript.

5. L69: Some researchers propose… this is not a sentence appropriate for a research article. Be specific on who proposes what with citations.

**Response:** Thank you for your valuable suggestion. We have revised the sentence in Lines 81–83 to specify the researchers and their proposed methods with appropriate citations.

6. L71 to L96 nice review.

**Response:** We appreciate your positive feedback.

7. L123: The text inside the figure can not be read. Make it larger (same font size as the text or at least same as the caption).

**Response:** Thank you for pointing this out. We have adjusted the font size within the figure to ensure that all text is clearly legible.

8. L162 Vit is already defined.

**Response:** We have revised the text to ensure that the abbreviation “Vit” is used consistently after its initial definition.

9. [L139–156]:

(a) Clarify whether patches are from the same date or synthesized (e.g., “cloudy patches from t1 and cloud-free from t0 or t2”).

(b) Add more information on cloud types in GF1-WHU dataset (e.g., vertical extent, stratification), not just "various conditions".

(c) Lacks detail on how image pairs are selected or validated. Describe what augmentations were used: flipping? rotation? brightness?

**Response:** Thank you for your valuable suggestions. We have carefully addressed each point as follows:

(a) As clarified in Lines 167–169 of the revised manuscript, all image patches referred to here are from the same date, ensuring temporal consistency..

(b) In Lines 176–181, we have added detailed descriptions of the cloud types included in the GF1-WHU dataset, including their vertical extent and stratification characteristics, to provide a clearer understanding of the dataset’s diversity in cloud conditions.

(c) We have provided additional details on the selection and validation of image pairs in Lines 167–170. Furthermore, the data augmentation techniques used in our study—such as flipping, rotation, and brightness adjustment—are now explicitly described in Lines 184–186.

10. [L160]: Add a sentence comparing ViT and CNNs specifically for SAR.

**Response:** To address this point, we have added a detailed comparison between ViT and CNNs in the context of SAR image global feature extraction in Lines 220–232. This addition helps clarify the rationale for using ViT by highlighting its advantages in handling SAR data.

11. [L224–226]: Be explicit about how reconstructed and non-reconstructed regions are defined in practice. Add a figure or schematic if possible.

**Response:** Thank you for the suggestion. We have explicitly defined the reconstructed and non-reconstructed regions in practice in Lines 266–270 of the revised manuscript. In addition, we have included a visual illustration in Figure 1, where the red box corresponds to the reconstructed region and the yellow box represents the non-reconstructed region.

12. [L228–229]: Clarify how spectral mapping is learned: supervised training? Patch loss metrics?

**Response:** Thank you for your comment. We have clarified the learning process of spectral mapping in Lines 274–279 of the revised manuscript. Specifically, the spectral mapping is learned through supervised training using the L1 loss function.

13. [L233–261]: The CGAN structure is described clearly, well done but needs justification of design choices (e.g., why no batch norm?).

**Response:** Thank you for your positive feedback. We have added a justification of the design choices for the CGAN generator, including the rationale for omitting batch normalization, in Lines 296–303 of the revised manuscript..

14. [L310–337]:

(a) Incomplete description of preprocessing steps.

(b) Lacks justification for scene selection (e.g., why Area A vs B?).

(c) No mention of data licensing, public access, or reproducibility..

**Response:** Thank you for your valuable suggestions. We have addressed each point as follows:

(a) A complete description of the data preprocessing steps has been added in Lines 397–402 of the revised manuscript.

(b) We have provided detailed justification for the scene selection in Lines 377–385.

(c) Information regarding data licensing, public accessibility, and reproducibility has been included in Lines 402–404.
